# Genetic variations of the A13/A14 repeat located within the *EGFR* 3′ untranslated region have no oncogenic effect in patients with colorectal cancer

**DOI:** 10.1186/1471-2407-13-183

**Published:** 2013-04-08

**Authors:** Nasrin Sarafan-Vasseur, David Sefrioui, David Tougeron, Aude Lamy, France Blanchard, Florence Le Pessot, Frédéric Di Fiore, Pierre Michel, Stéphane Bézieau, Jean-Baptiste Latouche, Thierry Frebourg, Richard Sesboüé

**Affiliations:** 1 Inserm U1079, Institute for Biomedical Research and Innovation, University of Rouen, 22 Boulevard Gambetta, CS 76183, Rouen Cedex, 76183, France; 2 Digestive Oncology Unit, Department of Hepato-Gastroenterology, Rouen University Hospital, 1 Rue de Germont, Rouen Cedex, 76031, France; 3 Department of Gastroenterology and Department of Oncology, Poitiers University Hospital, Laboratoire Inflammation Tissus Epithéliaux et Cytokines, University of Poitiers, Poitiers, EA 4331, France; 4 Laboratory of Tumor Genetics, University Hospital, 1 Rue de Germont, Rouen Cedex, 76031, France; 5 Department of Pathology, University Hospital, 1 Rue de Germont, Rouen Cedex, 76031, France; 6 Department of Genetics, Nantes University Hospital, Nantes, France

**Keywords:** Colorectal cancer, EGFR, Polymorphism, Microsatellite instability, Targeted therapy

## Abstract

**Background:**

The *EGFR* 3′ untranslated region (UTR) harbors a polyadenine repeat which is polymorphic (A13/A14) and undergoes somatic deletions in microsatellite instability (MSI) colorectal cancer (CRC). These mutations could be oncogenic in colorectal tissue since they were shown to result into increased EGFR mRNA stability in CRC cell lines.

**Methods:**

First, we determined in a case control study including 429 CRC patients corresponding to different groups selected or not on age of tumor onset and/or familial history and/or MSI, whether or not, the germline *EGFR* A13/A14 polymorphism constitutes a genetic risk factor for CRC; second, we investigated the frequency of somatic mutations of this repeat in 179 CRC and their impact on EGFR expression.

**Results:**

No statistically significant difference in allelic frequencies of the *EGFR* polyA repeat polymorphism was observed between CRC patients and controls. Somatic mutations affecting the *EGFR* 3′UTR polyA tract were detected in 47/80 (58.8%) MSI CRC *versus* 0/99 microsatellite stable (MSS) tumors. Comparative analysis in 21 CRC samples of EGFR expression, between tumor and non malignant tissues, using two independent methods showed that somatic mutations of the *EGFR* polyA repeat did not result into an EGFR mRNA increase.

**Conclusion:**

Germline and somatic genetic variations occurring within the *EGFR* 3′ UTR polyA tract have no impact on CRC genetic risk and EGFR expression, respectively. Genotyping of the *EGFR* polyA tract has no clinical utility to identify patients with a high risk for CRC or patients who could benefit from anti-EGFR antibodies.

## Background

Colorectal cancer (CRC) is the third most commonly diagnosed cancer in males and the second in females with 1.2 million new cases and 608,700 deaths estimated to have occurred worldwide in 2008 [1]. In its early stage, CRC represents a curable disease. However, 20–50% of patients with newly diagnosed CRC will develop secondary metastases (mCRC) [2]. A major advance in the treatment of mCRC has been achieved thanks to the development of targeted therapies. Accordingly, two antibodies, cetuximab and panatimumab, which selectively target the extracellular domain of the epidermal growth factor receptor (EGFR), have been approved for the treatment of metastatic diseases. The combination of these targeted molecules with conventional chemotherapy (5-FU, Irinotecan, Oxaliplatin) has led to significant improvement in response rate, progression free survival and overall survival in first line, as well as second or third line treatment of mCRC [3-8]. This efficiency constitutes a clinical evidence that activation of EGFR is oncogenic in CRC. However clinical trials have shown a high individual variability of response and outcome in mCRC patients, which has highlighted the need for identification of reliable markers predictive of response to treatment. The only molecular marker predictive of the response of the anti-EGFR mAbs, which has been unambiguously validated in mCRC by numerous studies, is the presence of *KRAS* activating mutations as a marker of resistance to anti-EGFR [9,10]. However the occurrence of *KRAS* mutations only accounts for 35–45% of non-responsive patients [11]. Remarkably, the mechanisms of EGFR activation in CRC have not been characterized in most of the patients. This contrasts with the situation observed in lung adenocarcinoma where the key mechanism of EGFR activation, underlying sensitivity to EGFR inhibitors, corresponds to activating mutations within the EGFR tyrosine kinase domain [12,13]. Indeed, in CRC, the amplification of *EGFR* resulting in overexpression and associated to sensitivity to anti-EGFR is detected in only 10–15% of CRC [14-17]. Overexpression of the EGFR ligands, amphiregulin and epiregulin, has been reported to be associated to sensitivity to anti-EGFR mAbs [18,19].

The *EGFR* gene contains within the 3′ untranslated region (UTR), 281 bp downstream from the stop codon, a polyadenine tract which is polymorphic (A13/A14). Mono or dinucleotide deletions within this polyA tract have been detected in colon cancer cell lines or CRC exhibiting microsatellite instability (MSI) [20]. These deletions have been shown to stabilize EGFR mRNA, to result in EGFR overexpression *in vitro* and to increase sensitivity to anti-EGFR antibodies in xenografts [20]. This prompted us to investigate, in CRC patients, the oncogenic impact of genetic variations affecting this regulatory region. To this aim, we used two complementary approaches: first, we determined, in a case control study, whether or not the germline *EGFR* A13/A14 polymorphism constitutes a genetic risk factor for CRC; second we investigated the frequency and impact of somatic mutations of this repeat in CRC.

## Methods

### Patients and samples

The germline *EGFR* A13/A14 polymorphism was investigated in a total of 429 CRC patients of French origin, corresponding to 4 groups: (1) Patients with CRC not selected on age of tumor onset or familial history (n = 179). This group, enriched in MSI tumors, corresponded to 80 MSI and 99 MSS CRC, as determined with a mononucleotide pentaplex panel [21]; (2) patients selected according to three different criteria suggestive of an increased genetic risk for CRC, but without detectable mutations in genes involved in Lynch syndrome or adenomatous polyposis (n = 62): (i) CRC before 61 years of age (or high-risk adenoma before 51 years of age) with a first-degree relative presenting with CRC; (ii) CRC before 51 years of age (or high-risk adenoma before 41 years of age); or (iii) multiple primitive colorectal tumors in the same patient, the first one diagnosed before 61 years of age if cancer or before 51 years of age if high-risk adenoma; (3) patients with Lynch syndrome harboring a mutation in one of the mismatch repair (MMR) genes (n = 100); (4) non selected sporadic CRC (n = 88). For the first group, germline *EGFR* A13/A14 polymorphism was genotyped from DNA extracted from paraffin embedded (FFPE) or frozen non malignant colorectal tissues. For the three others, DNA was extracted from peripheral blood samples after informed consent for genetic analyses had been obtained. DNA extraction from blood samples was performed using the FlexiGene kit (Qiagen), from FFPE samples, after manual macrodissection, using the Ambion RecoverAll kit (Applied Biosystems) and, from frozen samples, using the Nucleospin® Tissue kit (Macherey-Nagel EURL). *EGFR* allelic frequency in the general population was determined from 170 French controls, aged from 46 to 92 years.

Somatic mutations of the *EGFR* repeat were screened from FFPE or frozen tumor samples from the 179 CRC samples (group 1). For each patient, genomic DNA was extracted from paired tumor and normal colorectal tissues.

For each subject, a written consent had been obtained to perform genetic analyses either on blood or colorectal tissue and, in compliance with the Helsinki Declaration, the research programs on the molecular genetics of colorectal cancer had been approved by the ethics committee of Rouen and Nantes University hospitals.

### Genotyping of the *EGFR* 3’UTR polyA repeat

The *EGFR* 3’UTR polyA tract was amplified from 100 ng genomic DNA by fluorescent multiplex PCR targeting *EGFR* and *PCBD2*, as control (primers in Additional file 1). Amplification was performed in a final volume of 25 μl containing 1U of Diamond TaqDNA Polymerase® (Eurogentec) and 100 ng DNA, with the following conditions: after an initial step of denaturation at 95°C for 3 minutes, 24 PCR cycles consisting of denaturation at 94°C for 25 seconds, annealing at 58°C for 25 seconds, and extension at 72°C for 25 seconds, followed by a final extension step at 72°C for 25 seconds. Amplicons were separated on an ABI Prism 3100 DNA sequencer (Applied Biosystems), and the resulting fluorescence profiles were analysed using the Genescan software (version 3.7, Applied Biosystems). To ensure an accurate genotyping, we constructed molecular calibrators. To this end, the 3’UTR polyA tract was amplified from genomic DNA extracted from several cell lines obtained from the American Type Culture Collection (LGC Standards): MDA-MB-468 (HTB-132), NCI-H460 (HTB-177), DLD-1 (CCL-221) and SW48 (CCL-231). The amplicons were then cloned into the *Bam*H1-*Xho*1 site of pCDNA 3.1 (Clontech) and sequenced. Homozygous genotypes ranging from 10 to 14A were identified and heterozygous samples were obtained by mixing equal quantities of homozygous amplicons. Determination of the EGFR genotype was performed by superimposition of the profiles to that obtained from these molecular calibrators. Screening for EGFR somatic mutations was performed for each patient by superimposition of the profiles generated from tumor and paired non malignant CRC tissue.

### Measurement of EGFR expression

Frozen tumor tissue (TT) and paired normal tissue (NT) were collected from 21 CRC patients; normal tissue was obtained remote from the tumor, near the section boundary; for tumor tissue, an adjacent control fragment was embedded in paraffin, cut and stained with hemalun-eosin-safran to estimate the percentage of cancerous cells (on average 55%). Total RNA was extracted using the total RNA isolation Nucleospin RNA II® kit (Macherey-Nagel) following the manufacturer’s protocol. RNA quality was assessed by Experion® (BioRad) analysis. Total RNA (1.5 μg) was reverse transcribed using the SuperScript II reverse transcriptase for cDNA synthesis (Life Technologies) in a final volume of 40 μl at 40°C during 50 minutes in the presence of RNAse inhibitors (RNaseOUT™, Invitrogen). Two methods were used to accurately measure EGFR expression: quantitative RT-PCR was performed with the syber green gene expression assay for *EGFR* and, as internal control, *PGK* (primers in Additional file 1); reaction was performed with 100 ng of cDNA in the 7300 real time PCR system® apparatus (Applied Biosystem). The level of EGFR mRNA was calculated by relative quantitation using the comparative ΔΔCT threshold cycle method [22]. A semi quantitative RT-PCR (RT-QMPSF) assay was also developed, as previously described [23], and performed in a final volume of 50 μl using 2.5 μl of cDNA and 0.5 μl of Pwo DNA Polymerase® (Roche), using two endogenous control genes, *SF3A* and *PGK* (primers in Additional file 1). The PCR conditions were as follows: 95°C for 15 seconds followed by 27 cycles at 94°C for 15 seconds and 58°C for 30 seconds and 72°C for 45 seconds. Amplicons were separated on an ABI Prism 3100 DNA sequencer and the resulting fluorescence profiles were analysed using the Genescan software. The areas under curve (AUC) of amplicons were compared and normalized with the average AUC of control amplicons (*SF3A* and *PGK*).

### *In silico* analysis of mRNA secondary structures

Four web servers were used to modelize the EGFR mRNA secondary structure according to the number of adenines in the 3′ UTR polyA tract [24-27].

## Results

**Figure 1:**
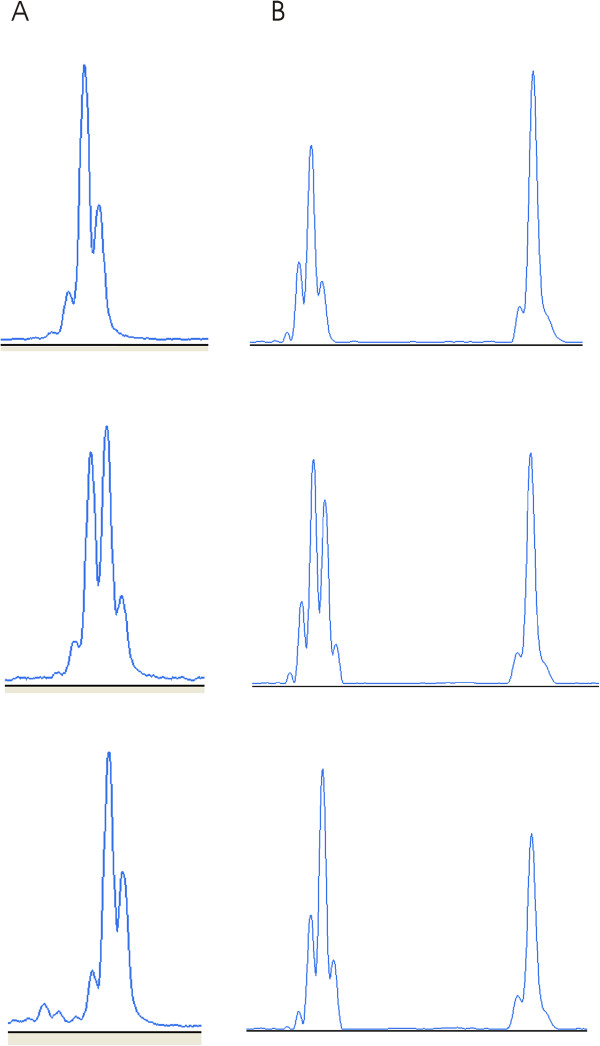
**Analysis of the germline *EGFR *3′UTR polyA repeat polymorphism, using fluorescent multiplex PCR. A**: Representative patterns obtained with cloned and sequenced amplicons corresponding to A13, A13/A14 and A14 repeats, from top to bottom. **B**: Representative patterns obtained with genomic DNA extracted from non malignant colorectal tissues corresponding to A13, A13/A14 and A14 repeats, from top to bottom; the peak to the right corresponds to the control (*PCBD2*) gene.

We genotyped the *EGFR* polyA repeat in non malignant colorectal tissue or blood from 429 patients with CRC corresponding to different groups of CRC patients selected or not on age of tumor onset and/or familial history and/or MSI. To ensure an acurate genotyping (Figure 1), we used, as calibrators, cloned *EGFR* polyA repeats the size of which had been determined by sequencing. Allelic frequencies observed in CRC patients and controls are given in Table 1. Allelic frequencies were in Hardy-Weinberg equilibrium in patients and controls. The frequency of the major allele (A13) was estimated in controls and patients to 76.5 and 72.8%, respectively. No statistically significant difference in allelic frequencies of the *EGFR* polyA repeat was observed between patients and controls and between each group of patients and controls (Table 1).

**Table 1:** **Allelic frequency of the *EGFR *3′UTR polyA repeat in CRC patients and controls**^**a**^

								
	**Controls**					**Patients**		
			**Group 1**		**Group 2**	**Group 3**	**Group 4**	**Total**
		**MSS**	**MSI**	**Total**				
Number	170	99	80	179	62	100	88	429
Age range (median)	46–92 (72)	66–88 (67)	25–99 (71)	25–99 (71)	46–62 (52)	19–66 (42)	70–92 (75)	25–99 (62)
A12	0.6% (0–2)	-	-	-	-	1% (0–4)	-	0.2%(0–1)
A13	76.5% (71–81)	70.7% (64–77)	73.7% (66–80)	72.1% (67–77)	74.2% (65–81)	73% (66–79)	73.3% (66–79)	72.8% (70–76)
A14	22.9% (18–28)	29.3% (23–36)	26.3% (20–34)	27.9% (23–33)	25.8% (18–34)	26% (20–33)	26.7% (20–34)	26.9% (24–30)
p value^b^		0.15	0.46	0.12	0.90	0.97	0.30	0.19

^a^ For each allelic frequency, confidence interval is given in brackets.

^b^ The p value in each patient group corresponds to the comparison with controls (chi-2 test).

**Figure 2:**
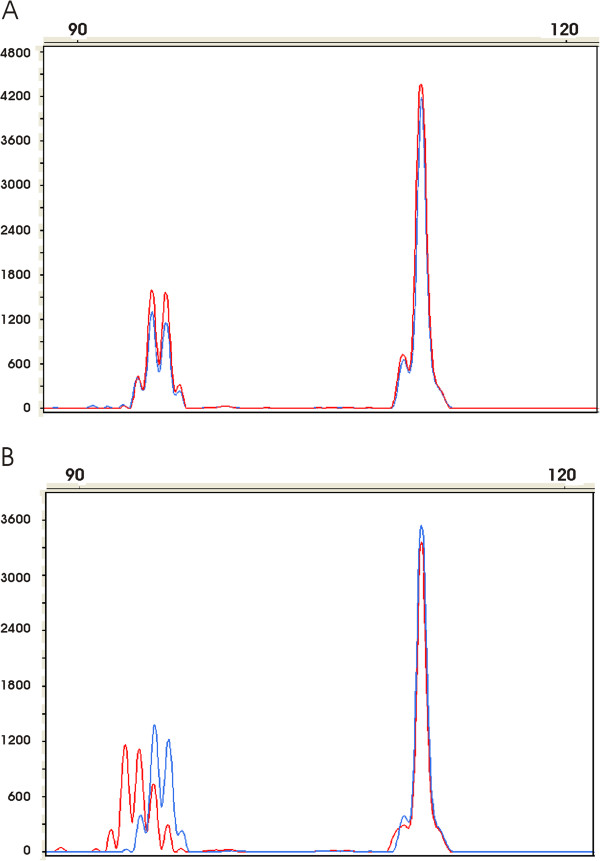
**Detection of *EGFR *3′UTR polyA tract somatic mutations, using fluorescent multiplex PCR.** The profile generated from malignant tissue (red) was superimposed on that obtained from distant non-malignant tissue (blue) after alignment of the control amplicons (peaks to the right corresponding to *PCBD2*). **A**: Pattern observed in a non mutated sample with A13/A14 genotype. **B**: Pattern observed in a mutated sample with A13/A14 genotype; notice in the tumor sample a shift of the peaks to the left corresponding to A11 and A12 repeats.

We then screened 179 patients with CRC for somatic mutations of the *EGFR* polyA repeats, by comparing, for each patient, the PCR profile obtained from tumor to that from paired non malignant tissue (Figure 2). As shown in Figure 2B, somatic *EGFR* polyA mutations could easily be detected by a clear shift of the EGFR fluorescent peak observed in tumors. In the 99 MSS CRC, we observed no somatic *EGFR* polyA mutation. In contrast, we detected an *EGFR* polyA mutation in 47/80 (58.8%) MSI CRC. The detected mutations always corresponded to adenine deletion and no gain was observed. The number of deletions ranged from 1 to 4 adenines and the total number of deletions observed on both alleles was: 1 (25.5%), 2 (27.7%), 3 (17%), 4 (12.8%), 5 (10.6%), 6 (2.1%), 7 (2.1%) and 8 (2.1%). There was no significant difference (chi-2 test, p = 0.70) in somatic mutation frequency (Table 2) in patients with A13/A13, A13/A14 and A14/A14 genotypes.

**Table 2:** **Frequency of somatic deletions observed in the *EGFR *3′UTR polyA tract according to the germline genotype in MSI patients**

**Germline genotype**	**Number of samples**	**Frequency of somatic deletions**
A13/A13	45	60.0 ± 2.1%
A13/A14	28	53.6 ± 3.5%
A14/A14	7	71.4 ± 12.6%

To address the specificity of somatic mutations affecting the *EGFR* 3′UTR polyA tract in MSI CRC, we evaluated in 10 MSI with *EGFR* mutations and 10 MSS CRC samples the frequency of mutations within two other 3′UTR polyA tracts sharing structure similar to that of the *EGFR*: a polyA(15) in *RAB31* (member RAS oncogene family) and a polyA(14) in *ATP6V1G1* (ATPase V1 subunit G1). In all MSI CRC samples with *EGFR* polyA tract mutations, we also found mutations of *RAB31* and *ATP6V1G1* polyA tracts, but no mutation was observed in MSS tumors.

We analyzed the potential impact of the *EGFR* 3′UTR polyA tract mutations on mRNA secondary structure through bioinformatics prediction. Successive deletions of adenine was not predicted to result in any significant alteration of the mRNA structure and, in particular, there was no modification of predicted binding sites for miRNAs (hsa-mir-146a/b, hsa-mir-133b, hsa-mir-7-1/2) or regulating proteins (HuR: AU-rich elements).

**Figure 3:**
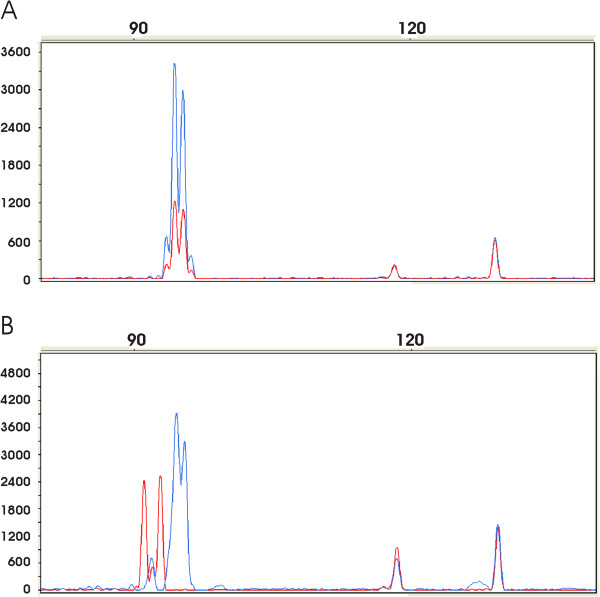
**Analysis of EGFR expression in non malignant and tumor colorectal tissues using fluorescent multiplex RT-QMPSF.** After adjustment on peaks corresponding to control genes (*PGK* and *SF3A*, peaks on the right), amplicons from normal (in blue) and tumor (in red) tissues are superimposed. **A**: Expression profiles in a non mutated sample from a patient with A13/A14 genotype. **B**: Expression profiles in a mutated sample from a patient with A13/A14 genotype; notice in the tumor sample a shift of the peaks to the left corresponding to A9 and A11 repeats.

We then determined the impact on EGFR expression of the somatic *EGFR* polyA tract mutations detected in MSI CRC, using real-time PCR quantitation of mRNA and RT-QMPSF (Figure 3). These two methods applied to 11 CRC with *EGFR* polyA mutation and 10 CRC without mutation yielded identical results (r = 0.75, see Additional file 2: Figure S1A). In 10/11 mutated and 10/10 non mutated samples, we observed, as illustrated in Figure 3, that the level of EGFR mRNA was lower in malignant tissue, as compared to paired normal tissue, although the difference was not significant. In the remaining mutated sample, we observed a slight increase (×1.1) of EGFR expression in tumor by comparison to normal tissue. There was no influence of the total number of adenine deletions on EGFR mRNA levels, even in a sample exhibiting up to 7 adenine deletions (see Additional file 2: Figure S1B). In 8 tumor samples harboring two *EGFR* alleles of different size and in 10 non malignant tissues from patients with a heterozygous genotype, we could compare the *EGFR* allelic expression by calculating the mRNA ratios corresponding to the short / long allele. In both cases, we did not observe an obvious allelic expression imbalance, but only a slight increase of expression of the short allele, as compared to the long one (mean 1.11 and 1.15, respectively).

Finally, we evaluated whether the germline *EGFR* polyA repeat polymorphism or mutational status in tumor influence the risk of tumor recurrence in 64 patients with a localized form of CRC (stage I, II and III) followed for at least two years. There was no difference in the percentages of recurrence according to the germline polyA polymorphism (p = 0.72), nor according to the existence or not of a somatic mutation (p = 0.72). In 18 patients with metastatic disease (stage IV) treated by anti-EGFR (cetuximab or panitumumab), the disease control rate was not influenced by the polyA tract polymorphism (p = 0.78).

## Discussion

We evaluated the biological impact, in patients with CRC, of germline or somatic genetic variations occurring within the *EGFR* 3′UTR polyA tract. First, we observed that the *EGFR* polyA allelic frequency in 429 CRC patients was similar to that observed in a control sample. Considering the genetic heterogeneity of CRC, we constructed the patient sample with 4 different groups selected or not on the basis of age of tumor onset or familial history or MSI status. The first group, composed of 179 CRC patients unselected on age of tumor onset or familial history, has been, on purpose, enriched in patients with MSI tumors, which had been shown in the original study of Yuan et al. [20] to exhibit a high rate of somatic *EGFR* mutations. The second group, constituted of 62 patients without detectable mutations within MMR or adenomatous polyposis genes but whose personal or familial history was suggestive of an increased genetic risk, was analyzed to determine whether or not the *EGFR* polyA polymorphism could act as a genetic risk factor for CRC. We also analyzed a series of 100 patients with Lynch syndrome to evaluate if the *EGFR* polyA polymorphism could act as a modifier risk factor in patients harboring a MMR gene mutation. Finally, the last group corresponded to 88 unselected sporadic CRC. In none of these groups, could a significant difference in *EGFR* allelic frequencies with controls be detected, suggesting that the *EGFR* 3′UTR polyA polymorphism does not modify the genetic risk for CRC. It could be argued that the size of the patient sample or that of the different groups was insufficient to detect a significant difference, but the allelic frequency between patients and controls were remarkably similar (Table 1). We also screened for somatic mutations of the *EGFR* polyA tract in the group of 179 CRC patients, whose genotypes had been characterized and found that somatic mutations, corresponding to deletions, were detected in 59% of the 80 MSI tumors but in none of the 99 MSS tumors. This confirms, on a larger sample, the results observed by Baranovskaya et al. [28], Yuan et al. [20] and Deqin et al. [29] who had reported, from a series of 40, 16 and 36 MSI CRC a mutation detection rate of 92.5%, 69% and 81%, respectively. Nevertheless, we obtained two results which argue against an oncogenic effect of these somatic mutations: first, the adenine deletions occurring in the 3′UTR polyA tract did not show any specificity with respect to *EGFR* since they could also be observed in 2 others genes not involved in CRC: *RAB31* and *ATP6V1G1*; therefore the high frequency of somatic *EGFR* polyA mutations reported in MSI tumors by other studies and this work probably reflects a particular sensitivity of mononucleotide tracts to defective DNA mismatch repair system, as recently reported for the polyT(20) tract of the *MT1X* gene [30]; second, we found that these mutations did not result into a significant increase of EGFR expression. In a study focused on the CA repeat located within the *EGFR* first intron, Baranovskaya et al. [28] have also observed, in agreement with our results, that EGFR expression was decreased in MSI CRC. In a sample composed of 16 MSI endometrial adenocarcinomas, Deqin et al. [29] have reported that tumors with *EGFR* polyA deletions exhibit a slight (1.6) but nevertheless not significant increase of EGFR expression, as compared to that without mutations. Our observation contrasts with results obtained by Yuan et al. [20]. Indeed, these authors had reported, in colon MSI cancer cell lines, that a deletion within the *EGFR* polyA tract increases *in vitro* the EGFR mRNA stability. In CRC patients, we observed that, in the majority of the tumor samples with somatic *EGFR* mutations (91%), the total level of EGFR mRNA was not increased but, in contrast, decreased and this result was obtained using two independent methods. The discrepancy observed between both studies highlights the need to confirm in clinical samples results previously obtained with cell lines which may not be representative of the complexity of gene regulation in clinical samples, because of the genetic drift occurring during *in vitro* culture.

## Conclusion

This study has raised several arguments showing that genetic variations affecting the *EGFR* polyA repeat are not involved in CRC development: (i) The *EGFR* polyA polymorphism does not constitute a genetic risk factor for CRC; (ii) somatic mutations of this repeat are commonly observed in MSI CRC, but their frequency reflects a sensitivity of this type of repeat to MSI and not a specific selective advantage; (iii) somatic *EGFR* polyA mutations do not result into an EGFR mRNA increase in colorectal tissue. Therefore, genotyping of the *EGFR* polyA tract has no clinical utility to identify patients with a high risk for CRC or patients who could benefit from anti-EGFR antibodies.

## Competing interests

The authors have no conflict of interest to declare.

## Authors’ contributions

Conception and design: NSV, TF, RS. Development of methodology: NSV. Acquisition of data: NSV, DS, DT, FLP, SB. Technical support: AL, FB. Analysis and interpretation of data: NSV, DS, FDF, PM, JBL, TF, RS. Study supervision: TF. Writing, review and/or revision of the manuscript: NSV, DS, TF, RS. All authors read and approved the final manuscript.

## Pre-publication history

The pre-publication history for this paper can be accessed here:

http://www.biomedcentral.com/1471-2407/13/183/prepub

## Supplementary Material

Additional file 1: Table S1 Primer sequences. Click here for file

Additional file 2: Figure S1 A: Correlation between RT-QMPSF (abscissa) and qRT-PCR (ordinate) results obtained on 21 CRC samples; mutated (♦) and non mutated (◊) samples. B: Ratio TT/NT obtained by qRT-PCR with respect to the total number of mutations (samples to the left correspond to non mutated tumor tissues); mutated (♦) and non mutated (◊) samples. Click here for file
