# Supplementary material for: Genetic variations of the A13/A14 repeat located within the EGFR 3′ untranslated region have no oncogenic effect in patients with colorectal cancer
Source: BMC Cancer. 2013 Apr 8;13:183. doi: 10.1186/1471-2407-13-183 (PMC3626788; doi:10.1186/1471-2407-13-183)
Supplement: Additional file 1: Table S1 — Primer sequences. [file 1471-2407-13-183-S1.doc]

Supplementary table 1 : primer sequences

|  | **Multiplex genomic PCR** | |
| --- | --- | --- |
| Target gene | Forward primer* | Reverse primer |
| ***EGFR*** | TTGTCCCTTTGAGCAGAAATTTAT | AAAACTCCAAGATCCCCAATCA |
| ***PCBD2*** | TCAGGCTTAGGGTAGAGGACAATG | TCTGCTTGTAGGGCAACTCG |
| ***ATP6V1G1*** | GGCTCAAGCAACATGTATATCAGTG | TTTAATCCCTTCCTTTCTTCAAAGA |
| ***RAB31*** | TGGAGGGGATGTAGTTGCATTT | TACACATAATAAGGCAGTAAGACTTTTGTA |
|  | **RT-QMPSF** | |
| Target gene | Forward primer* | Reverse primer |
| ***EGFR*** | TTGTCCCTTTGAGCAGAAATTTAT | AAAACTCCAAGATCCCCAATCA |
| ***SF3A*** | CAGAAACGGGCCTGAATT | CTTCCCTTCCTTGAACTCG |
| ***PGK*** | AGAGCCAGTTGCTGTAGAACT | CTGGGCCTACACAGTCCTTCA |
|  | **Quantitative RT-PCR** | |
| Target gene | Forward primer* | Reverse primer |
| ***EGFR*** | AACTGCGTGAAGAAGTGT | CTCCATCTCATAGCTGTCG |
| ***PGK*** | CTGGGCCTACACAGTCCTTCA | CTGGGCCTACACAGTCCTTCA |

*Forward primers were labelled with 6FAM.
