# Supplementary material for: Genetic variations of the A13/A14 repeat located within the EGFR 3′ untranslated region have no oncogenic effect in patients with colorectal cancer
Source: BMC Cancer. 2013 Apr 8;13:183. doi: 10.1186/1471-2407-13-183 (PMC3626788; doi:10.1186/1471-2407-13-183)
Supplement: Additional file 2: Figure S1 — A: Correlation between RT-QMPSF (abscissa) and qRT-PCR (ordinate) results obtained on 21 CRC samples; mutated (♦) and non mutated (◊) samples. B: Ratio TT/NT obtained by qRT-PCR with respect to the total number of mutations (samples to the left correspond to non mutated tumor tissues); mutated (♦) and non mutated (◊) samples. [file 1471-2407-13-183-S2.doc]

A

**0.0**

**0.2**

**0.4**

**0.6**

**0.8**

**1.0**

**1.2**

**1.4**


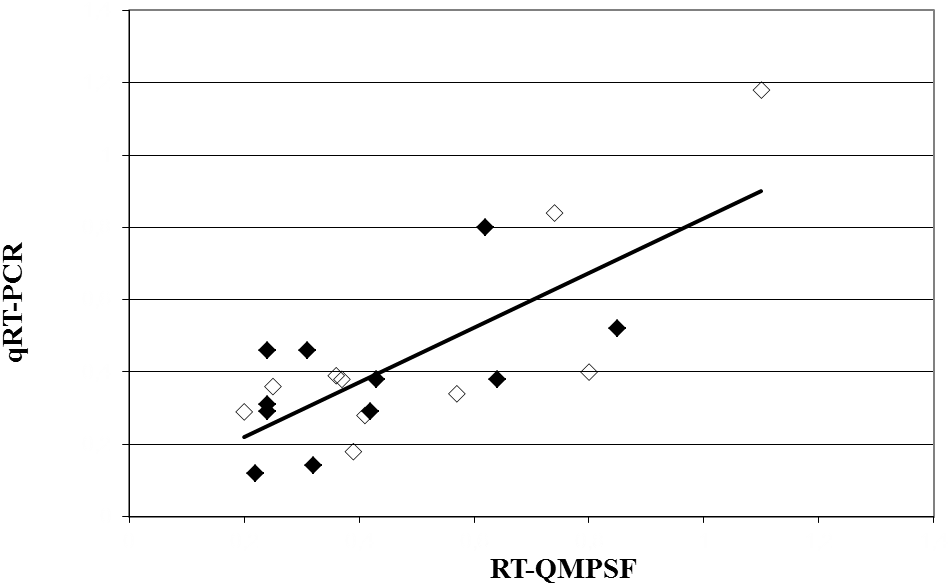


**0.0 0.2 0.4 0.6 0.8 1.0 1.2 1.4**

B


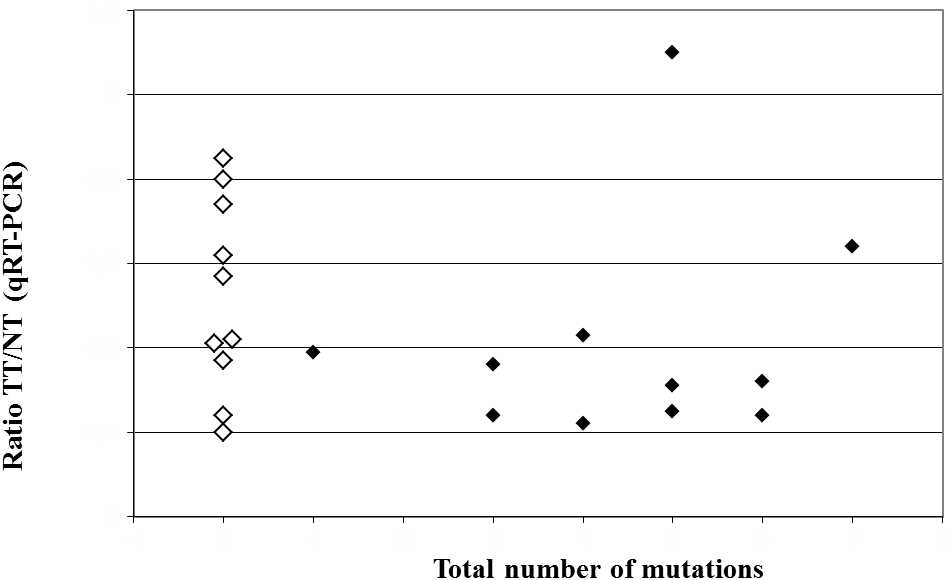


**0.0**

**0.2**

**0.4**

**0.6**

**0.8**

**1.0**

**1.2**

**0 1 2 3 4 5 6 7**

Supplementary figure 1: A: Correlation between RT-QMPSF (abscissa) and qRT-PCR (ordinate) results obtained on 21 CRC samples; mutated (◆) and non mutated (◇) samples. B: Ratio TT/NT obtained by qRT-PCR with respect to the total number of mutations (samples to the left correspond to non mutated tumor tissues); mutated (◆) and non mutated (◇) samples.
